# Superior Mesenteric Artery Syndrome in Systemic Lupus Erythematosus

**DOI:** 10.7759/cureus.42032

**Published:** 2023-07-17

**Authors:** India C Bradley, Bhavi Trivedi, Michael J Brockman, Mariam Hassan, Jose Sotelo, Tobi Okopie, Fatma Dihowm

**Affiliations:** 1 Internal Medicine, Texas Tech University Health Sciences Center El Paso, El Paso, USA

**Keywords:** systemic lupus erythromatosus, gastroenterology, sle, weight loss, autoimmune, superior mesenteric artery syndrome

## Abstract

Although the gastrointestinal (GI) manifestations of systemic lupus erythematosus (SLE) are relatively less reported, they are common and occur in approximately half of individuals with SLE. These symptoms vary and include, but are not limited to, oral ulceration, dysphagia, nausea, vomiting, diarrhea, abdominal pain, and intestinal perforation. Gastrointestinal manifestations are often triggered by an inciting event, such as an infection or the side effects of medication. This case report presents a rare GI-related SLE complication, namely superior mesenteric artery syndrome.

## Introduction

Systemic lupus erythematosus (SLE) is a chronic, multisystem autoimmune disease that causes widespread inflammation and tissue damage [1]. Patients with SLE often experience benign gastrointestinal (GI) manifestations, such as oral ulceration, nausea, vomiting, diarrhea, and abdominal discomfort [2]. However, SLE patients can present with more severe complications, including mesenteric vasculitis or ischemia, intestinal pseudo-obstruction, and hepatic and pancreatic involvement, too. Superior mesenteric artery (SMA) syndrome is an example of a more severe complication that is seldom reported in the scientific literature [3].

Superior mesenteric artery syndrome is a condition whereby the SMA compresses the third, or transverse, portion of the duodenum against the aorta [4]. Normally, fatty deposits in an otherwise healthy individual will increase the angle at which the SMA branches off the aorta. When the fat deposits become depleted, for example, secondary to poor oral intake or an SLE-induced hypermetabolic state, the branching angle becomes more acute and results in duodenal compression [4]. The symptoms of SMA syndrome are often nonspecific and present as epigastric pain, nausea, vomiting, and weight loss [5]. A diagnosis can be confirmed via imaging studies, and initial treatment is conservative with non-operative medical management [4, 5]. The authors report a unique case of a 25-year-old male with a history of SLE who exhibited significant weight loss, which was found to be due to a GI complication of SLE, namely SMA syndrome.

## Case presentation

A 25-year-old male with a past medical history of SLE presented to the emergency department with complaints of generalized weakness and fatigue. The patient also complained of persistent nausea, decreased appetite, worsening food intolerance, and diffuse myalgias that had been intensifying for one month prior to his presentation. His poor oral intake resulted in a 40-pound weight loss in one year. He also complained of chest tightness, shortness of breath on exertion, and Raynaud’s phenomenon.

The patient was diagnosed with SLE 14 months prior to presentation after he began to have severe weakness and decreased range of motion. To his knowledge, the patient had no other coexisting autoimmune conditions or mixed connective tissue disorders. Home-prescribed medications included methotrexate, hydroxychloroquine, folic acid, and prednisone. The patient self-discontinued hydroxychloroquine eight months prior, methotrexate four months prior, and prednisone and folic acid two to four weeks prior to presentation. Per the patient, he followed up with his rheumatologist inconsistently, his last visit being six to eight months prior, and failed to follow up due to mood-related symptoms.

On arrival, the patient was afebrile with a temperature of 36.9°C, hypotensive with a blood pressure of 97/60 mmHg, and tachycardic with a heart rate of 101 bpm. The physical examination was remarkable for a severely underweight patient at 36.3 kg with a body mass index (BMI) of 11.9 kg/m2. He appeared cachectic, dehydrated, and had poor dentition. His muscle strength was graded at four out of five in the upper and lower extremities, bilaterally, with decreased active range of motion of his fingers and tenderness to palpation secondary to sclerodactyly, calcinosis, and ulcerations of the finger digits. He presented with a flat affect and made poor eye contact. A gastrointestinal examination revealed a sunken abdomen that was soft, non-tender, and non-distended, with normal bowel sounds.

The laboratory results are presented in Table 1.

**Table 1:** The patient's laboratory parameters at presentation

	Presentation	Reference range
White blood cell count	3.76	4.50 - 11.00 (x10^3^/uL)
Hemoglobin	13.4	12.0 - 16.0 (g/dL)
Hematocrit	38.2	38.0 - 47.0%
Platelets	161	150 - 450 (x10^3^/uL)
Sodium	133	135 - 145 (mmol/L)
Potassium	4.4	3.5 - 5.1 (mmol/L)
Serum creatinine	0.4	0.66 - 1.25 (mg/dL)
Urea	12	9 - 20 (mg/dL)
Bicarbonate	24	22 - 30 (mmol/L)
Aspartate transaminase	248	17 - 59 (IU/L)
Alanine transaminase	96	0 - 50 (IU/L)
Urine protein	20	0 - 14 (mg/dL)
Urine creatinine	49	20 - 320 (mg/dL)

The antinuclear antibody (ANA) test was highly positive, with a 1:5120 titer and a positive anti-dsDNA 1:10 titer. Other laboratory results showed low complement levels, elevated C-reactive protein (CRP) at 1.72 mg/dL and erythrocyte sedimentation rate (ESR) at 89 mm/hr, and positive Smith, histone, and ribonucleoprotein (RNP) antibodies. Other anti-inflammatory workups were negative, including anti-centromere, RNA polymerase III IgG antibody (Ab), anti-topoisomerase I Ab, anti-Sjögren’s-syndrome-related antigen A, and cardiolipin Ab.

**Figure 1:**
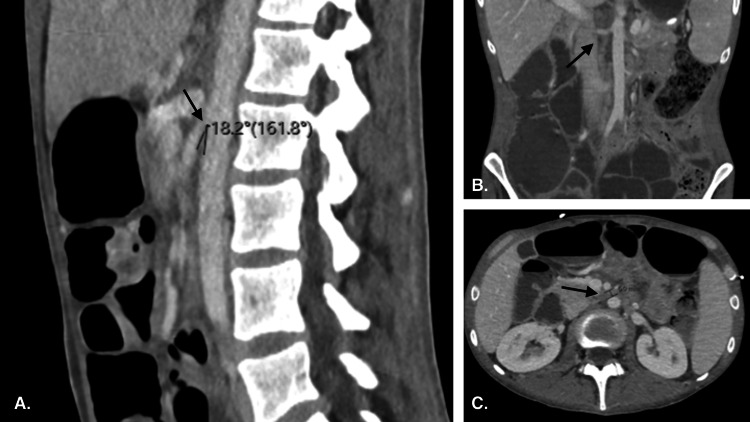
A contrast-enhanced computed tomography (CT) of the thorax, abdomen, and pelvis is shown. The CT revealed a loss of fat pad between the SMA and aorta, resulting in duodenal compression with a shortened SMA-aortic angle (arrows). 1A: sagittal view; 1B: coronal view; 1C: axial view SMA: superior mesenteric artery

On admission, the patient was placed on a regular diet with high-protein shakes per dietary recommendations. Maintenance fluids were initiated, and electrolytes were closely monitored for refeeding syndrome and replenished as needed. Two days after admission, the patient continued to complain of poor appetite, severe nausea, and intractable vomiting when attempting to eat. A computed tomography (CT) of the abdomen and pelvis with contrast revealed dilation of the stomach and the first and second portions of the duodenum with large amounts of air and fluid present. There was a narrowing of the SMA branching angle from the aorta secondary to a paucity of subcutaneous fat, suggestive of SMA syndrome (Figure 1).

A kidney, ureter, and bladder x-ray was performed, which showed intense stool burden but no obstruction. An esophagogastroduodenoscopy was performed by the gastroenterology (GI) team, which showed grade D esophagitis.

After the diagnosis of SMA syndrome, a nasogastric tube was subsequently placed to provide intermittent suction for decompression. The patient was encouraged to increase oral intake and was placed on a regular diet with the addition of Ensure® Pro high-protein shakes three times a day per nutritional recommendations. A daily dose of mirtazapine was initiated to increase his appetite and elevate his mood. The patient was restarted on his home medication, hydroxychloroquine, and given amlodipine and topical nitrates for Raynaud’s phenomenon and sclerodactyly. The nasogastric tube was removed, and the patient had some clinical and laboratory improvement on discharge five days later, with decreased vomiting and a gradual increase in oral intake. On discharge, the patient was tolerating a soft diet with mildly tolerable nausea. The patient was discharged home with a referral to follow-up outpatient with a rheumatologist, GI, and psychiatrist. Medications on discharge included hydroxychloroquine, mirtazapine, amlodipine, pantoprazole, and folic acid. The patient was noted to have significant improvement in his GI symptoms, particularly the abdominal pain, and nutritional status at his three-week outpatient follow-up. At his four-month follow-up, the patient was noted to have gained 7 kg of weight and had been following up regularly with GI, psychiatry, and rheumatology.

## Discussion

Vague symptoms in patients with SLE should warrant further workup, which may lead to our presented diagnosis. Gastrointestinal symptoms that may be encountered in SLE patients are precipitated by a multitude of factors, including medication side effects, infection, peptic ulcers, and mesenteric vasculitis [6, 7]. Here, we present the case of a young man stricken with SLE, which ultimately led to depression and weight loss. In our patient, the cause of poor oral intake was unusual and resulted in severe malnourishment with excessive weight loss. The third part of the duodenum typically passes between the aorta and the SMA. The normal SMA-aorta angle is usually 38-65 degrees due to the presence of the mesenteric fat pad [5]. If this fat pad were to decrease, it could lead to obstruction of the duodenum and SMA syndrome. Superior mesenteric artery syndrome is often reported secondary to trauma, surgery, malignancy, burns, and other disorders associated with extreme weight loss, including spinal injury and anorexia. Superior mesenteric artery syndrome as a complication of SLE with significant weight loss, however, has only been reported once in the virtually accessible literature. Unlike our patient, this previously reported case presented a patient with an acute exacerbation of lupus, leading to weight loss and SMA syndrome [3].

Systemic lupus erythematosus (SLE) and SMA syndrome are distinct medical entities and do not have a direct causal relationship. Although the exact mechanism by which SLE causes SMA syndrome remains incompletely understood, it is suspected that poor oral intake due to nausea or medication side effects propagates malnourishment and, eventually, these same symptoms will stem from SMA syndrome instead. Given that the signs or symptoms of SMA syndrome significantly overlap with the GI-related complaints in SLE patients, the authors suspect that SMA syndrome is significantly underdiagnosed in the SLE population.

The treatment for SMA syndrome can range from non-interventional management to interventional management with surgery [8]. Non-operative management includes bowel rest with possible nasogastric tube insertion, IV fluid therapy with electrolyte repletion, analgesics, and antiemetics as needed. If the patient is not clinically improving after 72 hours or worsening, more interventional management might include exploratory laparotomy, endoscopic intervention for decompression and detorsion, and stool evacuation [8]. In this case, our patient was found to have clinical improvement with non-operative management based on oral intake and did not require any further interventions.

**Figure 2:**
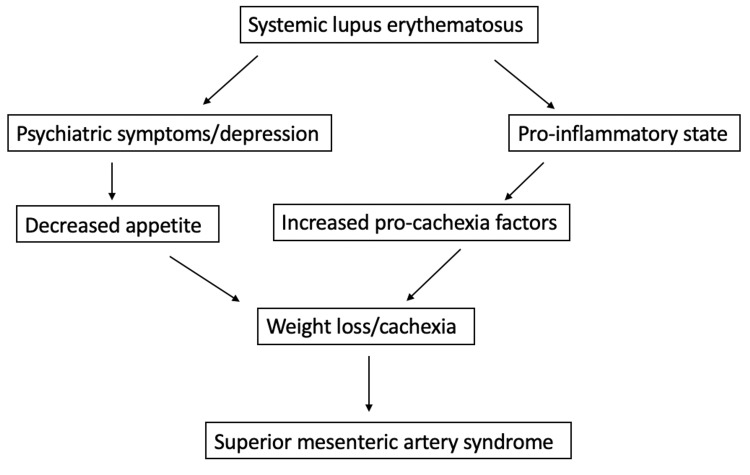
A schematic diagram showing the possible relationship between SLE and SMA syndrome

Weight loss, for example, is seen among SLE patients with an incidence of around 10%-20%, but it is also implicated in SMA syndrome [9]. According to the American College of Rheumatology, depression and cognitive dysfunction are the earliest signs of neuropsychiatric symptoms in SLE patients [10, 11]. A decreased appetite and subsequent weight loss fall under the umbrella of depression. Recent studies have also shown that cachexia, or involuntary weight loss, is an under-recognized symptom in patients with SLE [12]. The mechanisms underlying this phenomenon are currently under research, with multiple theories involving pro-inflammatory mediators [12]. Other studies have shown that lupus may interfere with the production or usage of leptin, causing decreased appetite and increased energy use, leading to the compression of the SMA [13]. Hence, the authors postulate that the patient’s nausea, worsening food intolerance, and medication noncompliance led to weight loss, which in turn prompted SMA syndrome through excessive fat loss. The patient’s flat affect was suggestive of depression and contributed to an ongoing cycle of further loss of appetite and weight loss. This vicious cycle is depicted in Figure 2.

## Conclusions

With the varying presentation of GI symptoms in patients with SLE, a thorough evaluation is necessary, which may lead to our presented diagnosis of SMA syndrome. Treatment of SMA syndrome in SLE may involve management of the underlying autoimmune disorder or surgical intervention to alleviate the compression of the SMA. A multidisciplinary approach involving gastroenterology, rheumatology, and surgery is required for the complete management of SMA syndrome in patients with SLE. Treatment would require medical interventions such as the initiation of immunosuppressive therapy to control inflammation of the vessels, nutritional support, and symptomatic management with antiemetic medications to assist with nausea that these patients often suffer from. Ultimately, surgery may be required for patients who do not respond to medical management. Here, we present a case of SLE-induced SMA, an uncommon presentation managed successfully by a multidisciplinary approach.
